# Peripheral blood lymphocyte subpopulations in patients with bipolar disorder type II

**DOI:** 10.1038/s41598-019-42482-6

**Published:** 2019-04-10

**Authors:** Krzysztof Pietruczuk, Katarzyna A. Lisowska, Karol Grabowski, Jerzy Landowski, Wiesław J. Cubała, Jacek M. Witkowski

**Affiliations:** 1 Department of Pathophysiology, Medical University of Gdansk, Faculty of Medicine, Gdańsk, Poland; 2 Department of Psychiatry, Medical University of Gdansk, Faculty of Medicine, Gdańsk, Poland

## Abstract

We investigated the phenotype of peripheral blood lymphocytes of patients with bipolar disorder type II in different phases of the disease in order to check whether there are specific changes in the immune parameters. Lymphocytes subpopulations were analyzed *ex vivo* with flow cytometry in patients in euthymic, depression or hypomanic phase of the disease and compared with healthy controls. All BD patients were characterized by lower percentage of CD3^+^CD4^+^ and CD3^+^CD8^+^ cells compared with healthy people. But only patients in depression and remission had higher percentage of B cells (CD19^+^ cells) compared with healthy people. The percentage of CD4^+^CD25^+^ and CD8^+^CD25^+^ cells was decreased in patients in hypomanic phase compared with healthy control. Patients in remission were characterized by increased concentrations of IL-6 and IL-10 and decreased level of TNF in blood serum. Significant correlations between immunologic parameters and the results of Hamilton or Young scale have also been found. Our results demonstrate that there are significant differences in lymphocyte subpopulations which depend on the phase of the disease the patient is currently in.

## Introduction

Psychoneuroimmunology is an interdisciplinary branch of biomedical science that studies the relationship between the nervous system, endocrine and immunology and their interaction with the psyche (mainly stress and its consequences)^1^. The ability of these systems to communicate with each other is facilitated by the production of various chemical mediators and the presence of their receptors on different cells^2,3^. Lymphocytes and macrophages have noradrenergic, cholinergic and peptidergic receptors on their surfaces which enables to create “neuroimmunological synapses” between nervous and immune cells^4^. Lymphocytes can bind different neurotransmitters including norepinephrine, dopamine, adrenaline, GABA, acetylcholine, and peptides (substance P, vasoactive intestinal peptide, somatostatin, neuropeptide Y)^4–6^. These substances control most of the functions of the immune cells such as differentiation, proliferation, antigen expression and cytokine production^4,6–8^.

On the other hand, cytokines regulate not only the nervous system, but are also produced by neurons and glial cells^2,4,9^. Some, like interleukin 1 (IL-1) or tumor necrosis factor α (TNF-α), are involved in the fever development and loss of appetite that accompanies inflammation^10^. Ye *et al*. reported that plasma levels of IL-6 and TNF-α are significantly higher in depressed patients than in healthy people^11^. Treatment with antidepressants significantly reduces plasma levels of IL-6 and TNF-α^12–14^. At the same time, the level of IL-10, which is neuroprotective and anti-inflammatory cytokine, increases in patients who are being treated with antidepressant drugs belonging to different groups (serotonin re-uptake inhibitors, tricyclics, and heterocyclic)^15,16^.

Bipolar disorder (BD) is a chronic mental disorder in which the patient experiences both depression and mania (BD type I) or hypomania (BD type II)^17^. There are also other subtypes and forms of bipolar disorder referred as “bipolar spectrum”^18^. Treatment of bipolar disorder consists of pharmacotherapy and psychotherapy. Types of interventions and drugs used in treatment depend on the type of disorder, its phase and severity. Main therapeutic goals are the reduction of acute symptoms of depression or hypomania/mania and maintaining of stable state (euthymic state).

Studies have shown that compared with healthy people, BD patients present increased plasma levels of IL-4, IL-10 as well as IL-6 and TNF-α^19^. Barbosa *et al*. demonstrated increased levels of soluble receptor for TNF-α type 1^20,21^. BD patients also are characterized by the reduced percentage of total T lymphocytes (CD3^+^ cells)^22,23^ as well as population of cytotoxic T lymphocytes (CD3^+^CD8^+^ cells) compared with healthy people^22^. Barbosa *et al*. also observed that BD patients exhibit higher percentage of activated CD4^+^CD25^+^ T cells^22^.

It should be noted that in the majority of the above cited studies, BD patients qualified for the study were in a state of euthymia. It would be interesting to check if the phase of the disease affects the immune parameters. Therefore, we investigated the proportions of main lymphocyte subpopulations in patients with BD type II depending on the phase of disease they were in and compared them with those observed in healthy people. We also analyzed whether there are differences in the percentages of lymphocytes expressing activation CD25 antigen which is related to IL-2 dependent proliferation, cytokine production and expansion of lymphocyte populations^24–26^. We investigated the concentration of cytokines (IL-6, TNF-α, IL-17A, IL-10) in the serum of BD patients and healthy individuals. Also, we correlated immunologic parameters with the results of Hamilton or Young scales.

## Results

**Figure 1:**
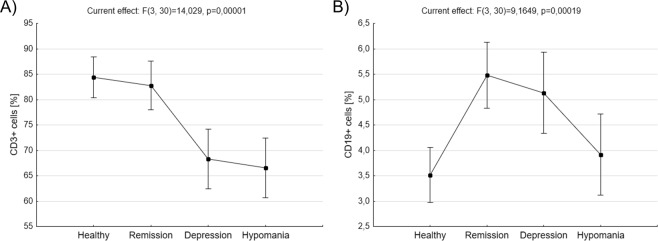
Comparison of proportions of CD3^+^ and CD19^+^ cells *ex vivo*. Figures show percentages of CD3^+^ cells (**A**) and CD19^+^ cells (**B**) in healthy people, BD patients in remission, depression or hypomania. Middle points show means and vertical bars represent 0.95 confidence intervals, ANOVA test.

Percentage of two main lymphocyte populations, that is T lymphocytes (CD3^+^ cells) and B lymphocytes (CD19^+^ cells), was compared between BD patients in remission, depression, hypomania, and healthy people. BD patients in depression phase of the disease were characterized by significantly lower percentage of CD3^+^ cells compared to healthy control (68,33 ± 2,87 *vs*. 84, 38 ± 1,95, p = 0,002477, ANOVA with post-hoc Tukey test) and patients in remission (68,33 ± 2,87 *vs*. 82, 77 ± 2,35, p = 0,006811, ANOVA with post-hoc Tukey test, Fig. 1A). BD patients in hypomania phase of the disease also were characterized by significantly lower percentage of CD3^+^ cells compared to healthy control (66,50 ± 2,87 *vs*. 84, 38 ± 1,95, p = 0,000823, ANOVA with post-hoc Tukey test) and patients in remission (66,50 ± 2,87 *vs*. 82, 77 ± 2,35, p = 0,002150, ANOVA with post-hoc Tukey test, Fig. 1A). BD patients in remission had significantly higher percentage of CD19^+^ cells compared to healthy control (5,47 ± 0,32 *vs*. 3,52 ± 0,26, p = 0,000929, ANOVA with post-hoc Tukey test) and patients in hypomanic phase (5,47 ± 0,32 *vs*. 3,92 ± 0,39, p = 0,039806, ANOVA with post-hoc Tukey test, Fig. 1B). Also, BD patients in depression had significantly higher percentage of CD19^+^ cells compared to healthy control (5,13 ± 0,39 *vs*. 3,52 ± 0,26, p = 0,031415, ANOVA with post-hoc Tukey test, Fig. 1B). No difference has been found in the percentage of CD19^+^ cells between patients in hypomania and healthy people.

**Figure 2:**
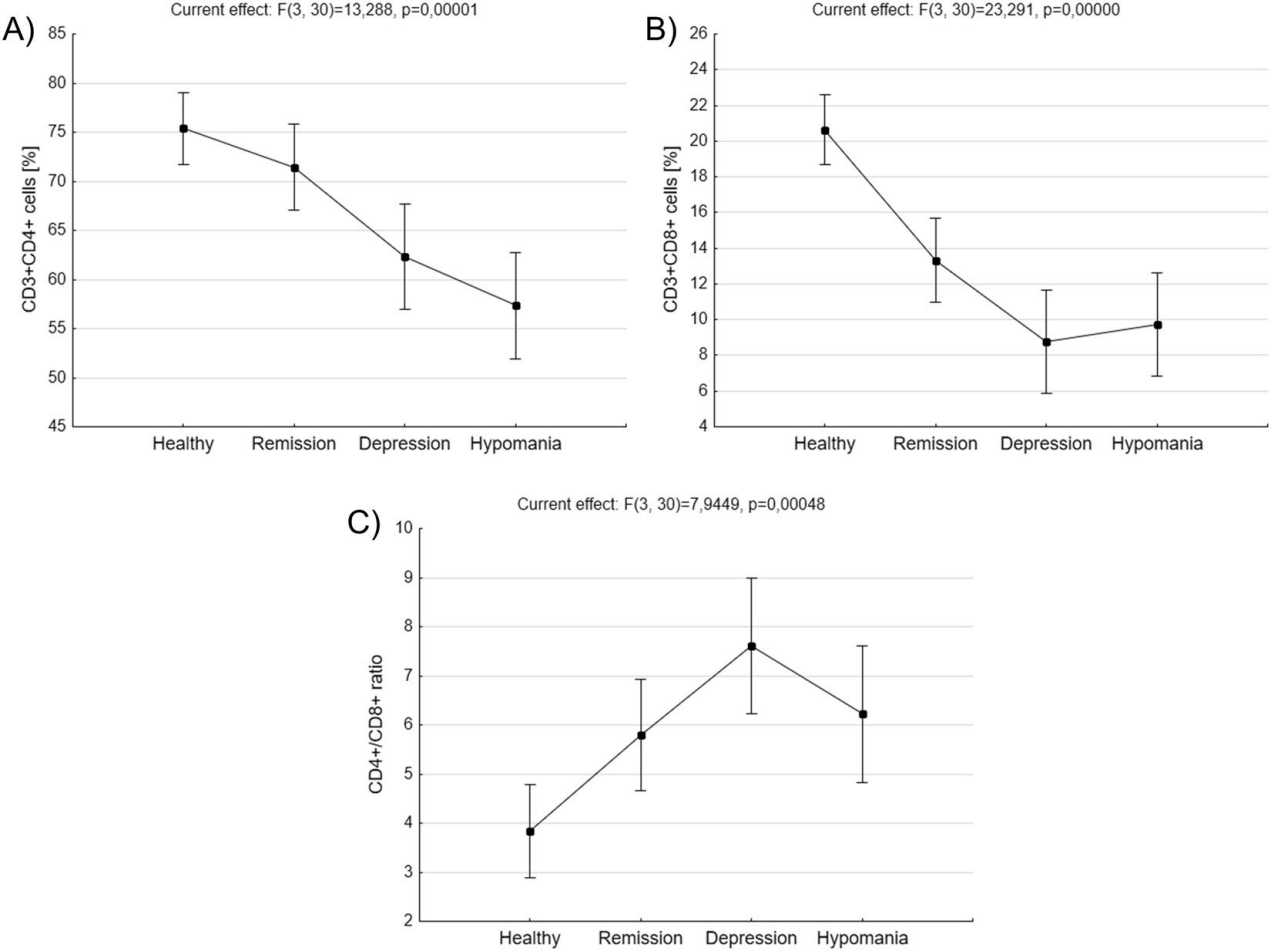
Comparison of main CD3^+^ subpopulations *ex vivo*. Figures show percentages of CD3^+^CD4^+^ cells (**A**), CD3^+^CD8^+^ cells (**B**) as well as CD4^+^/CD8^+^ ratio (**C**) in healthy people, BD patients in remission, depression or hypomania. Middle points show means and vertical bars represent 0.95 confidence intervals, ANOVA test.

Patients in depression had significantly lower percentage of CD3^+^CD4^+^ cells compared to healthy control (62,33 ± 2,64 *vs*. 75,38 ± 1,79, p = 0,007804, ANOVA with post-hoc Tukey test, Fig. 2A). BD patients in hypomania phase of the disease also were characterized by significantly lower percentage of CD3^+^CD4^+^ cells compared with healthy control (57,33 ± 2,64 *vs*. 75,38 ± 1,79, p = 0,000343, ANOVA test with post-hoc Tukey test) and patients in remission (57,33 ± 2,64 *vs*. 71,44 ± 2,15, p = 0,003788, ANOVA test with post-hoc Tukey test, Fig. 2A). No difference has been found in the percentage of CD3^+^CD4^+^ cells between patients in hypomania and depression.

Compared with healthy people, BD patients in depression were characterized by significantly lower percentage of CD3^+^CD8^+^ cells (8,77 ± 1,41 *vs*. 20,62 ± 0,95, p = 0,000166, ANOVA with post-hoc Tukey test, Fig. 2B). Patients in hypomania phase of the disease had also significantly decreased percentage of CD3^+^CD8^+^ cells compared with healthy control (9,72 ± 1,41 *vs*. 20,62 ± 0,95, p = 0,000186, ANOVA with post-hoc Tukey test, Fig. 2B). Euthymic patients also had significantly lower percentage of CD3^+^CD8^+^ cells compared with healthy control (13,22 ± 1,15 *vs*. 20,62 ± 0,95, p = 0,000677, ANOVA with post-hoc Tukey test, Fig. 2B). The significant difference in the CD4^+^/CD8^+^ ratio was observed only in patients with depression compared to healthy people (7,61 ± 0,68 *vs*. 3,84 ± 0,46, p = 0,002689, ANOVA with post-hoc test, Fig. 2C). CD4^+^/CD8^+^ ratio was 6,23 ± 0,68 in BD patients in hypomania and 5,79 ± 0,55 in euthymic patients.

**Figure 3:**
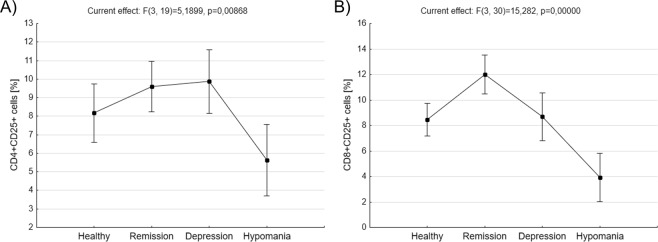
Comparison of T lymphocytes with the expression of CD25 antigen *ex vivo*. Figures show percentages of CD4^+^CD25^+^ cells (**A**) and CD8^+^CD25^+^ cells (**B**) in healthy people, BD patients in remission, depression or hypomania. Middle points show means and vertical bars represent 0.95 confidence intervals, ANOVA test.

We also analyzed subpopulations of T lymphocytes with the expression of CD25 (activated T cells). BD patients in hypomanic phase of the disease had significantly decreased percentage of CD4^+^CD25^+^ cells compared to patients in remission (5,64 ± 0,92 *vs*. 9,59 ± 0,65, p = 0,030309, ANOVA with post-hoc Tukey test) or depression (5,64 ± 0,92 *vs*. 9,87 ± 0,82, p = 0,019462, ANOVA with post-hoc Tukey test, Fig. 3A). They also had significantly decreased percentage of CD8^+^CD25^+^ cells compared to patients in remission (3,92 ± 0,93 *vs*. 12 ± 0,75, p = 0,000163, ANOVA with post-hoc Tukey test) or depression (3,92 ± 0,93 *vs*. 8,68 ± 0,93, p = 0,005437, ANOVA with post-hoc Tukey test) as well as healthy people (3,92 ± 0,93 *vs*. 8,44 ± 0,63, p = 0,008647, ANOVA with post-hoc Tukey test, Fig. 3B). Additionally, BD patients in remission has significantly increased percentage of CD8^+^CD25^+^ cells compared to healthy people (12 ± 0,75 *vs*. 8,44 ± 0,63, p = 0,011947, ANOVA with post-hoc Tukey test).

Drugs that patients received (lithium or valproic acid) did not affect the above immunological parameters in the study group (data not shown).

**Figure 4:**
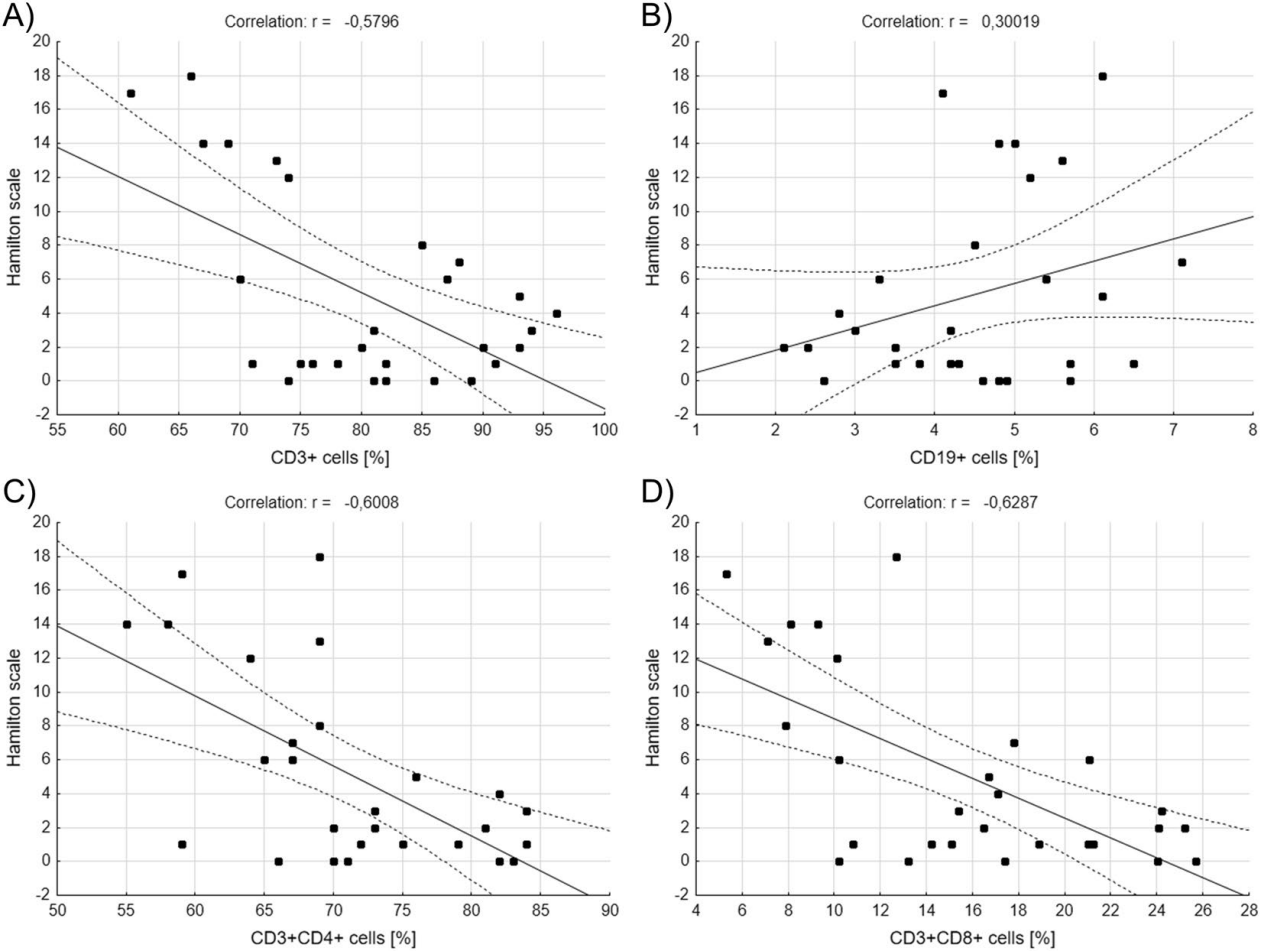
Correlations between main lymphocytes’ subpopulations *ex vivo* and results of Hamilton Rating Scale. Figures show correlations between percentages of CD3^+^, CD19^+^, CD3^+^CD4^+^ or CD3^+^CD8^+^ and the results of Hamilton Rating Scale for Depression (**A**–**D**), Pearson correlation.

We also correlated percentages of lymphocyte subpopulations with the results of Hamilton Rating Scale for Depression or Young Rating Scale for Mania. We included in the calculations healthy people, BD patients in remission and patients with depression for Hamilton scale (Fig. 4). And so, we found out that there are significantly negative correlations between Hamilton scale results and percentages of CD3^+^ (r = −0,579642, r^2^ = 0,335985, p = 0,001227, Pearson’s correlation, Fig. 4A), CD3^+^CD4^+^ (r = −0,600828, r^2^ = 0,360994, p = 0,000722, Pearson’s correlation, Fig. 4C) and CD3^+^CD8^+^ (r = −0,628726, r^2^ = 0,395296, p = 0,000339, Pearson’s correlation, Fig. 4D) cells that is, the higher score the subject received, the lower the percentage of lymphocytes was. No correlation has been found between Hamilton scale results and percentage of CD19^+^ cells (Fig. 4B). Also, there was significant positive correlation between Hamilton scale results and CD4^+^/CD8^+^ ratio (r = 0,581207, r^2^ = 0,337801, p = 0,001181, Pearson’s correlation, data not shown).

**Figure 5:**
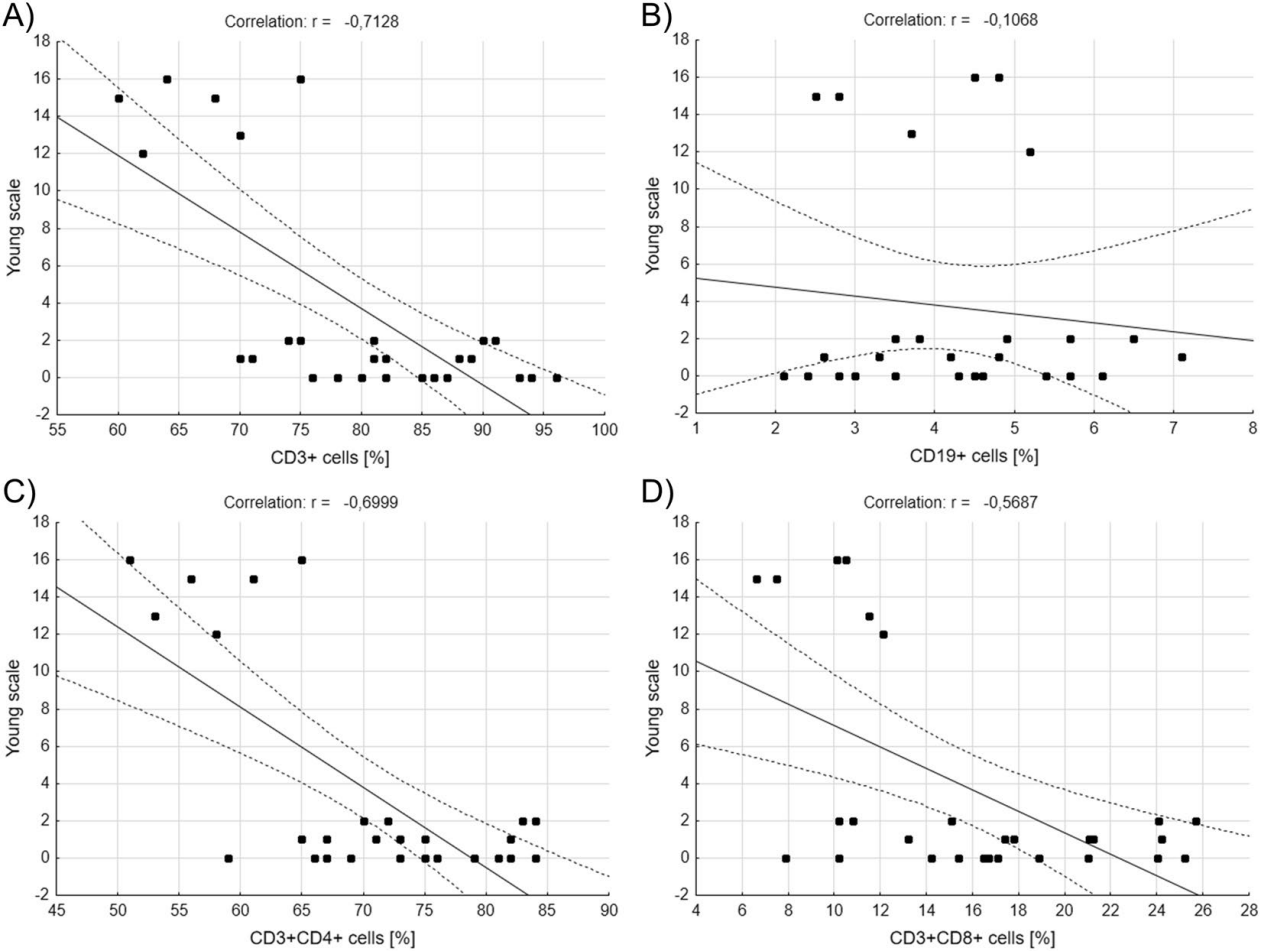
Correlations between main lymphocytes’ subpopulations *ex vivo* and results of Young Rating Scale. Figures show correlations between percentages of CD3^+^, CD19^+^, CD3^+^CD4^+^ or CD3^+^CD8^+^ and the results of Young Rating Scale for Mania (**A**–**D**) obtained by healthy people and BD patients, Pearson correlation.

Similar correlations were performed for Young scale – this time healthy people, BD patients in remission and patients with hypomania were included in the calculations (Fig. 5). We saw a significantly negative correlations between Young scale results and percentages of CD3^+^ (r = −0,712800, r^2^ = 0,508084, p = 0,000021, Pearson’s correlation, Fig. 5A), CD3^+^CD4^+^ (r = −0,699857, r^2^ = 0,489800, p = 0,000034, Pearson’s correlation, Fig. 5C) and CD3^+^CD8^+^ (r = −0,568740, r^2^ = 0,323465, p = 0,001589, Pearson’s correlation, Fig. 5D) cells. No correlation has been found between Young scale results and percentage of CD19^+^ cells (Fig. 5B). Also, there was significant positive correlation between Hamilton scale results and CD4^+^/CD8^+^ ratio (r = 0,384955, r^2^ = 0,148190, p = 0,043090, Pearson’s correlation, data not shown).

**Figure 6:**
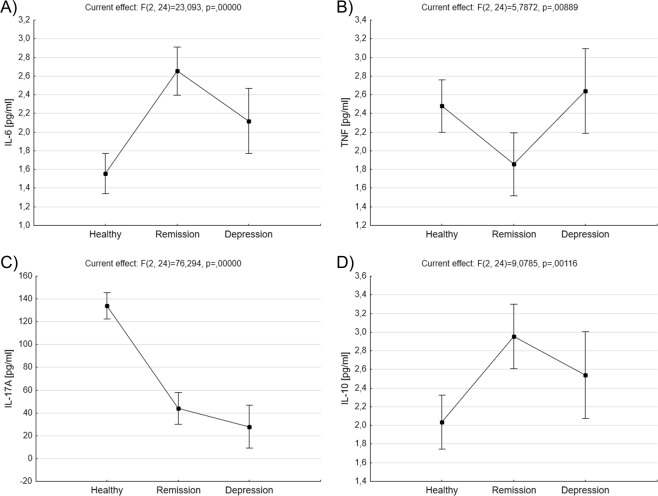
Comparison of cytokines’ levels in serum samples. Figures show concentrations (pg/ml) of IL-6 (**A**), TNF (**B**), IL-17A (**C**) and IL-10 (**D**) in serum samples of healthy people and BD patients. Middle points show means and vertical bars represent 0.95 confidence intervals, ANOVA test.

The concentration of cytokines (IL-6, TNF-α, IL-17A, IL-10) was measured in the serum of healthy subjects and BD patients in remission or depression. Euthymic patients were characterized by significantly increased level of IL-6 compared with healthy control (2,65 ± 0,12 *vs*. 1,55 ± 0,1, p = 0,000132, ANOVA with post-hoc Tukey test, Fig. 6A). TNF-α concentration was significantly decreased in patients in remission compared to healthy people (1,85 ± 0,16 *vs*. 2,48 ± 0,13, p = 0,032135, ANOVA with post-hoc Tukey test) and patients in depression (1,85 ± 0,16 *vs*. 2,64 ± 0,22, p = 0,046543, ANOVA with post-hoc Tukey test Fig. 6B). Compared with healthy people BD patients in remission were characterized by significantly decreased concentration of IL-17A (44 ± 6,76 *vs*. 134,15 ± 5,63, p = 0,000129, ANOVA with post-hoc Tukey test, Fig. 6C). Similar thing concerned patients in depression - mean concentration of IL-17A was 28 ± 9,07 *vs*. 134,15 ± 5,63 (p = 0,000129, ANOVA with post-hoc Tukey test, Fig. 6C). BD patients in remission had also significantly increased level of IL-10 compared with healthy control (2,95 ± 0,16 *vs*. 2,03 ± 0,13, p = 0,002037, ANOVA with post-hoc Tukey test, Fig. 6D). No difference has been found in the level of IL-10 cells between patients in depression and healthy people.

## Discussion

The results of this study confirm that there are changes in lymphocyte subpopulation *ex vivo*, which strongly depend on the phase of bipolar disorder the patient is currently in. First of all, BD patients who were depressed or hypomanic were characterized by a low percentage of CD3^+^ cells compared with healthy people and patients who were in remission, which was accompanied by decrease in the percentages of CD3^+^CD4^+^ and CD3^+^CD8^+^ cells. These changes were significantly associated with the results of Hamilton (for the depressed patients) or Young (for the patients in hypomanic phase) scales, which means that the higher score the patient received (more depressed or maniac he was), the lower the percentage of T lymphocytes was. Low percentage of T lymphocytes, especially cytotoxic ones (CD3^+^CD8^+^ cells), has already been reported by Barbosa *et al*.^22^. Unlike us, authors did not observed decrease in the percentage of helper lymphocytes (CD3^+^CD4^+^ cells). The difference in these observations can be easily explained; Barbosa *et al*. included only euthymic BD patients into their studies. Meanwhile we have shown that the percentage of both CD3^+^CD4^+^ and CD3^+^CD8^+^ cells was decreased in depressed and hypomanic patients, whereas in patients in remission only cytotoxic cells were still lowered. We believe that this is very important clinical observation, which proves that during the active phase of disease all T lymphocytes “disappear” from patient’s blood, probably because they migrate to the brain. Since the classical role of cytotoxic T cells is to mediate host defense against the intracellular infectious agents (especially viruses), decrease of CD3^+^CD8^+^ cells observed even during the remission, according to Barbosa *et al*., could support the hypothesis of a close relationship between BD and infection caused by viruses^22^. Compared with the general population, BD patients are more predisposed to some infectious diseases, particularly hepatitis B or C^27^ and human immunodeficiency virus infection^28^. An increase in the incidence of certain viral diseases in BD patients actually may be the result of immune disorders in the course of the disease, especially since studies have failed to demonstrate the presence of viral genome in patients with psychiatric disorders.

According to study of Barbosa *et al*.^22^, euthymic BD patients are characterized by higher percentages of activated (CD4^+^CD25^+^) cells *ex vivo*. Meanwhile, we demonstrated that this is true only for CD8^+^CD25^+^ cells, which would also support the thesis of a close relationship between BD and viral infections. Knijff and colleagues showed that BD patients do not present any differences in activated T lymphocytes (expressing CD25^+^) regardless the phase of the disease^29^. However, they were studying expression of CD25 after *in vitro* stimulation which makes difficult to compare their results with the results obtained *ex vivo* by Barbosa *et al*. Interestingly, in our study we have demonstrated that patients in hypomania had a low percentage of CD4^+^CD25^+^ and CD4^+^CD25^+^ cells *ex vivo* compared with other groups of patients and healthy people, which seems to be their characteristic feature.

Another interesting observation was that patients in remission or depression in opposition to hypomanic patients had higher percentage of B cells *ex vivo* compared with healthy people. So far, there have been no reports of disturbances in the humoral response in BD. However, there are observations showing that euthymic BD patients are characterized by a higher concentration of IL-10^30^, a cytokine, which plays an important role in B cell activation and stimulates immunoglobulin class switching. We measured the level of IL-10 in the serum samples of our patients in depression and remission and saw higher, compared with samples from healthy people, concentrations of IL-10 in euthymic patients. Unfortunately, we didn’t have any samples from patients in hypomania to compare and confirm a relationship between IL-10 level and B cell percentage but we believe that the increase of B cell percentage observed *ex vivo* in these patients could be explained by high level of IL-10. The question remains why patients in remission have immune changes similar to those seen in depressed patients. Maybe it is related to the condition that preceded the remission of the patients who were qualified for our study. The ideal way to answer this question would be to observe the same BD patient in all stages of the disease and analyze whether immunologic changes are related to the particular phase.

Both patients in depression and remission had lower concentration of IL-17A compared with healthy people, which we believe results from high concentration of IL-10 – as demonstrated by Gu *et al*.^31^, IL-10 is a negative suppressor of Th17 cell differentiation. Meanwhile, concentration of IL-6 differentiated patients in remission from depressed patients. IL-6 is known to stimulate B cell differentiation into plasma cells and it increases immunoglobulin G production^32^. Therefore, an increase of IL-6 could be another factor contributing to B cell increase observed in euthymic patients. At the same time, patients in remission were characterized by decreased TNF concentration compared with healthy people and patients in depression. It may be related to the fact that euthymic BD patients have higher blood levels of soluble TNF receptor (sTNFR)^20^ that can bind TNF. Generally, data regarding the production of IL-6, TNF and IL-10 are contradictory with some studies showing decreased production of these cytokines, while others present no significant differences^11–14,19^. It seems that several things should be taken into account when comparing immunological results: (1) which patients were qualified for the study (euthymic, depressed or manic), (2) the effect of drugs on some of the parameters which cannot be excluded, and (3) whether the cytokine concentrations were measured in serum or in cell culture supernatants after mitogenic stimulation. According to Modabbernia *et al*.^19^ as well as Barbosa *et al*.^33^, due to the above-mentioned factors it is almost impossible to draw definite conclusions on specific immune markers for BD. Our team has recently demonstrated that lithium and valproic acid can influence T lymphocytes stimulated with monoclonal anti-CD3 antibody; both drugs can protect lymphocytes from apoptosis^34^. However, their inhibitory effect on lymphocyte proliferation is visible only at toxic doses. In this study, drugs that BD patients received (lithium or valproic acid) did not affect either percentages of lymphocyte subpopulations *ex vivo* or concentration of cytokines. Modabbernia *et al*. demonstrated similar results but for IL-2, IL-4 and INF-γ^19^. Their meta-analysis also have shown that BD patients had a near significant increase in IL-6 concentrations compared with healthy people probably because patients in mania showed elevated trends of IL-6^19^. Results we obtained for IL-10 concentrations seem to be similar to those shown by other authors^19^. However, according to Modabbernia *et al*.^19^, BD patients seem to rather show significantly higher TNF-α values in patients than healthy control, which our results do not confirm. Discrepancies in the results mean that further research is needed preferably performed in the same patients at different stages of the disease.

These results demonstrate that there are differences in subpopulations of T and B lymphocytes that possibly dependent on the current phase of bipolar disorder, which was also demonstrated by observed correlations between some immunologic parameters and the results of Hamilton Rating Scale for Depression or Young Rating Scale for Mania. In our opinion, immune responses can depend on the form of bipolar disease presentation. Another possibility, more provocative, is that maybe changes in the immune responses can trigger course of the disease towards depression or hypomania. However, we must admit that at the moment we cannot draw definitive conclusions on this subject. First of all, the number of examined patients should be increased in each phase of the disease. Furthermore, it would be a good idea to expand the immunological panel, which would allow to find other features differentiating the phase of bipolar disorder. However, the most interesting results can be provided by observation of the patient for one or even two years during various phases of the disease, to correlate the observed changes in the parameters with the patient’s mental state. This could be the answer to the question whether immune changes are truly specific to specific phases of the disease.

## Methods and Material

### Patients and healthy control

14 healthy (8 female and 6 male) people (mean age 39,45 ± 10,4) and 22 (11 female and 11 male) bipolar disease type II patients (mean age 37,91 ± 7,4) participated in the study. All participants underwent basic physical examination and psychiatric interview in Clinic of Adult Psychiatry of the Medical University of Gdansk. Patients were diagnosed with the Structured Clinical Interview for DSM-V^35^. Mental state of patients was assessed using Hamilton Depression Rating Scale^36^, and Young Mania Rating Scale^37^. 10 euthymic patients were included in the study, 6 patients with depression and 6 patients in mania phase (Table 1). BD patients with depression achieved significantly higher results in Hamilton scale compared to healthy people (14,66 ± 0,99 *vs*. 1,92 ± 0,67, p = 0,000132, ANOVA with post-hoc Tukey test) and patients in remission (14,66 ± 0,99 *vs*. 3,22 ± 0,81, p = 0,000132, ANOVA test with post-hoc Tukey test). Patients in hypomania achieved significantly higher results in Young scale compared to healthy people (14,5 ± 0,43 *vs*. 0,77 ± 0,29, p = 0,000132, ANOVA with post-hoc Tukey test) and patients in remission (14,5 ± 0,43 *vs*. 0,66 ± 0,35, p = 0,000132, ANOVA test with post-hoc Tukey test). 10 patients were treated with lithium carbonate and 12 received valproic acid. The time of therapy with lithium or valproic acid was average 14,43 ± 2.02 months.

**Table 1:** Basic characteristic of BD patients and healthy people.

	Patients (n = 22)			Healthy people (n = 14)
	Remission (M/F)	Depression (M/F)	Mania (M/F)	
Age (years)	37,91 ± 7.4			39,45 ± 10,4
Sex (M/F)	11/11			6/8
Phase of Bipolar Disorder	10 (6/4)	6 (2/4)	6 (3/3)	—
Lithium carbonate	10			—
Valproic acid	12			—
Hamilton Rating Scale for Depression	3,22 ± 0,81	14,66 ± 0,99	—	1,92 ± 0,67
Young Rating Scale for Mania	0,66 ± 0,35	—	14,5 ± 0,43	0,77 ± 0,29

Exclusion criteria for the control group were: diagnosed mental disorders and/or incidence of mental illness in the family. Exclusion criteria for both patients and healthy people included: autoimmune disorders, chronic inflammatory disorder, diabetes and allergies. None of the patients admitted to drug addiction. A few people admitted to occasional alcohol consumption.

All participants were informed about the purpose of the study and gave their written informed consent. The study was approved by Independent Bioethical Committee for the Scientific Research of the Medical University of Gdansk. All methods were performed in accordance with the relevant guidelines and regulations.

### Collecting peripheral blood and serum

3 ml of venous peripheral blood was collected from patients and healthy controls in tubes containing EDTA as the anti-coagulant after overnight fasting for the cytometric analysis of lymphocytes’ subpopulations. 5 ml of blood was collected into anticoagulant-free tubes in order to collect serum for the assessment of cytokine concentrations. Serum samples were stored at −80 °C.

### Determination of lymphocyte subpopulations

Samples of 50 μl per tube blood were transferred for staining with monoclonal antibodies and red blood cells (RBCs) lysis. RBCs were lysed with buffer containing 0,8% NH_4_Cl and 0,1% KHCO_3_. Cells were then washed with PBS (phosphate buffered saline) buffer and stained with: APC-Cy7-conjugated anti-CD3, PE-Cy5-conjugated anti-CD4, V500-conjugated anti-CD8, PE-Cy7-conjugated anti-CD25 and APC-H7-conugated anti-CD19 antibodies (Becton Dickinson, USA) for 30 minutes at 4 °C in the dark. After this time cells were washed with PBS and suspended in 200 µl of PBS buffer for flow cytometric analysis.

Quantitative fluorescence analysis was performed with FACSVerse (Becton Dickinson, USA). 10000 lymphocytes (based on their forward and side scatter gating) were acquired from each sample. Cytometric data were analyzed with FlowJo X 10.0.7 (Tree Star; USA).

### Cytokine measurements in the serum

Cytometric Bead Array (CBA™, BD Biosciences, USA) was used to estimate the level of IL-6, TNF-α, IL-17A, IL-10 cytokines in patients’ serum. Concentrations of cytokines were analyzed with the use of Becton Dickinson CBA software.

### Statistical analysis

Statistical analysis was done with the Statistica version 10 (StatSoft, Inc., USA). The Kolmogorov-Smirnov and Lilliefors tests were used for testing normality. The significance tests were chosen according to data distribution with the level of significance p < 0.05.
